# Recent Advances in Drug Delivery Strategies for High-Risk BCG-Unresponsive Non-Muscle Invasive Bladder Cancer: A Brief Review from 2018 to 2024

**DOI:** 10.3390/pharmaceutics16091154

**Published:** 2024-08-30

**Authors:** Feng Qu, Saloni Darji, David H. Thompson

**Affiliations:** Bindley Bioscience Center, Department of Chemistry, Multi-Disciplinary Cancer Research Facility, Purdue University, West Lafayette, IN 47907, USA; qu99@purdue.edu (F.Q.); darji@purdue.edu (S.D.)

**Keywords:** Bacillus Calmette–Guérin-unresponsive, non-muscle invasive bladder cancer, intravesical therapy, bladder preservation, gene therapy

## Abstract

High-risk BCG-unresponsive non-muscle invasive bladder cancer (NMIBC) is a condition that is typically treated with Bacillus Calmette–Guérin (BCG) therapy. Unfortunately, NMIBC is characterized by high recurrence, with a significant percentage of BCG patients ultimately requiring radical cystectomy. As a consequence, the development of effective new therapies to avoid RC has become a rapidly evolving field to address this unmet clinical need. To date, three biologics—Keytruda, Adstiladrin, and Anktiva—have been approved by the FDA, and multiple drug modalities, particularly gene therapies, have shown promising results in clinical trials. Advances in drug delivery strategies, such as targeted delivery, sustained release, and permeabilization of protective layers, are critical in overcoming the challenges posed by therapeutic intervention in bladder cancer. This review focuses on high-risk BCG-unresponsive NMIBC therapies that have been or are currently being investigated in clinical trials, offering a broad overview of the delivery system designs and up-to-date clinical outcomes that have been reported as of July 2024. It aims to inform the development of future drug delivery systems for second-line therapies in high-risk BCG-unresponsive NMIBC.

## 1. Introduction

**Figure 1:**
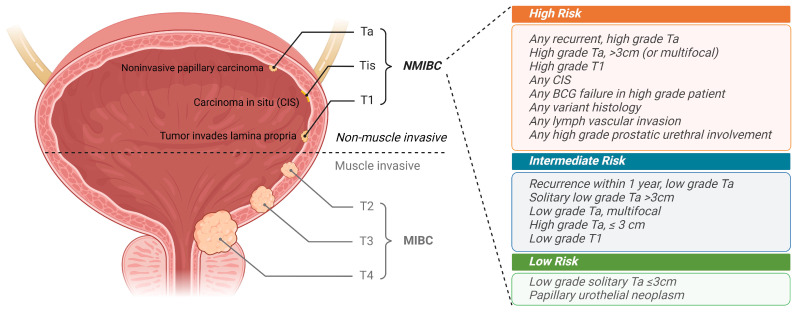
Staging of primary tumors in bladder cancer and risk stratification for NMIBC. Ta: noninvasive papillary carcinoma of low malignant potential, Tis: carcinoma in situ (CIS), and T1: tumor invades lamina propria are classified as NMIBC. T2: tumor invades muscularis propria, T3: tumor invades perivesical tissue/fat, and T4: tumor invades prostate, uterus, vagina, pelvic wall, or abdominal wall are classified as MIBC. Risk stratification for NMIBC includes low-risk (patients meet all criteria), intermediate-risk (patients meet any criteria), and high-risk (patients meet any criteria).

In the United States, an estimated 82,290 new cases of bladder cancer and 16,710 deaths due to the disease were projected for 2023 [1]. For newly diagnosed bladder cancer patients, a transurethral resection of the bladder tumor (TURBT) is performed to remove the visible tumor. Based on the depth of bladder wall invasion observed in the TURBT-removed tissue, the pathological stages of Ta, Tis, and T1 are classified as non-muscle invasive bladder cancer (NMIBC), which accounts for 75% of the initial bladder cancer diagnoses [2]. NMIBC is further stratified into low, intermediate, and high-risk groups based on the pathological stage, tumor grade, size, multiplicity, presence of carcinoma in situ (CIS), and recurrence history (Figure 1) [3]. The gold standard therapy for high-risk NMIBC patients is intravesical instillation of BCG, involving weekly induction for 6 weeks and 3 weekly maintenance doses at 3, 6, 12, 18, 24, 30, and 36 months [4]. However, BCG therapy fails in about one-third of the NMIBC patients. This challenge was previously described as BCG failure and subclassified into BCG refractory, resistant, relapsing, and intolerant [5]. Early studies have extensively investigated intravesical chemotherapeutic agents, such as valrubicin, gemcitabine, mitomycin C (MMC), and docetaxel, as salvage therapies for BCG failure. However, the overall outcomes were not promising, with 70–80% of patients experiencing recurrence within two years [6]. This is partially attributed to the varying entry criteria between trials at that time. To accurately identify patients unlikely to benefit from further BCG therapy, the US FDA has recently defined BCG-unresponsive disease as meeting at least one of the following: “(1) persistent or recurrent CIS alone or with recurrent Ta/T1 disease within 12 months of completion of adequate BCG therapy; (2) recurrent high-grade Ta/T1 disease within 6 months of completion of adequate BCG therapy; (3) T1 high-grade disease at the first evaluation following an induction BCG course” [7]. For those high-risk BCG-unresponsive NMIBC patients, the standard of care involves radical cystectomy (RC). However, many patients are ineligible for RC or seek to preserve their bladder, which significantly increases their risk of progression to muscle-invasive bladder cancer (MIBC) with a worse survival prognosis. Thus, there is an urgent need for effective bladder-preserving therapies that can help these patients avoid RC.

**Figure 2:**
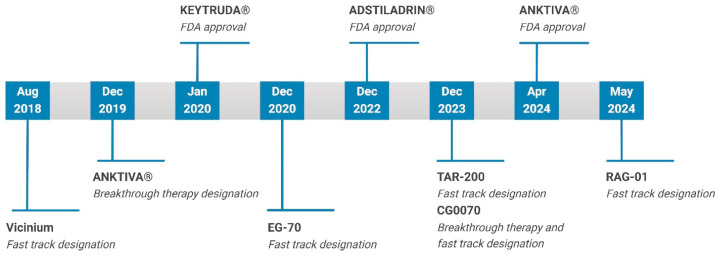
Timeline of key developments in the treatment of high-risk BCG-unresponsive NMIBC.

To address the unmet clinical need for high-risk BCG-unresponsive NMIBC patients, the FDA issued pivotal guidance in February 2018 that clarified trial entry criteria, acceptable trial designs, and primary efficacy endpoints [7]. This regulatory clarity has significantly impacted the development of bladder-preserving therapies. In the past five years (2020–2024), three new biologics have been approved by the FDA to treat high-risk BCG-unresponsive NMIBC with CIS with or without papillary tumors (Figure 2). Additionally, several novel treatments have shown promising results in late-stage clinical trials and are expected to gain approval soon. This review will focus on the drug delivery strategies utilized in these therapies, highlighting multiple modalities that have been investigated in clinical trials or approved by the FDA since the 2018 guidance.

## 2. Route of Administration (RoA)

**Figure 3:**
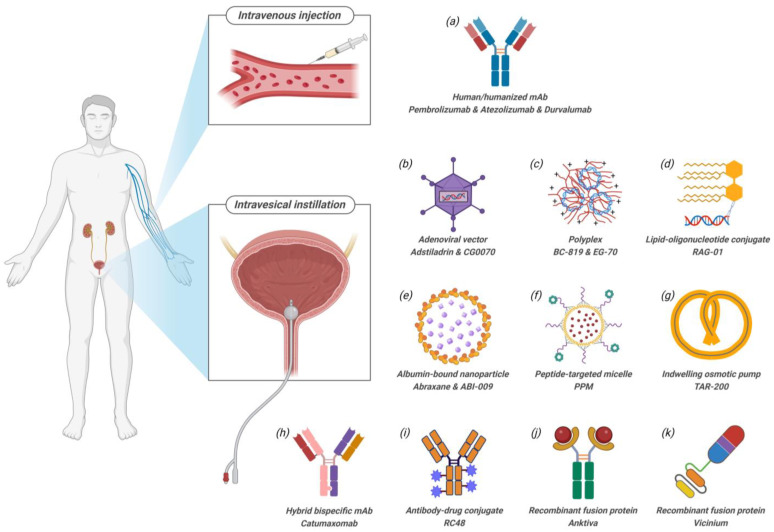
Primary RoAs and drug modalities for the treatment of high-risk BCG-unresponsive NMIBC.

The bladder is a hollow organ that stores urine, lined by the urothelium, where bladder cancer can develop. Widely employed approaches for NMIBC treatment involve intravesical instillation and intravenous injection (Figure 3). Intravesical instillation provides high local drug concentrations at the site, a limited retention time before voiding, and minimized potential systemic side effects. The urothelium consists of basal, intermediate, and superficial umbrella cells, with fully differentiated umbrella cells forming a high-resistance barrier on the apical surface. Additionally, the surface of the urothelium is coated by the glycosaminoglycan (GAG) layer, a thick mucus layer composed of glycoproteins and proteoglycans such as heparan sulfate, chondroitin sulfate, and hyaluronic acid. These components are negatively charged and highly hydrophilic, entraining water molecules firmly within the GAG layer [8,9]. While the umbrella cells and the GAG layer together form a mucosal surface that protects the bladder urothelium from indwelling toxic metabolites within the urine and bacterial infections that may occasionally be present in urine, it also poses a significant obstacle for intravesical drug delivery. 

Intravenous injection is another viable RoA that generally offers better patient compliance but increases the risk of systemic toxicity. It is important to note that the definition of complete response (CR), the primary efficacy endpoint in BCG-unresponsive NMIBC clinical trials, differs between intravenous and intravesical therapies. Intravenous/systemic therapies are expected to be effective for the entire urinary tract, so patients will not be considered CR if malignant lesions are present in the upper tract or prostatic urethra, even if the cystoscopy is negative. In contrast, for intravesical therapies, malignant urine cytology with cancer in the upper tract or prostatic urethra can be considered CR if the cystoscopy and random bladder biopsies are negative [7].

## 3. Small Molecule Drug Formulations

### 3.1. Peptide-Targeted Micelles

**Figure 4:**
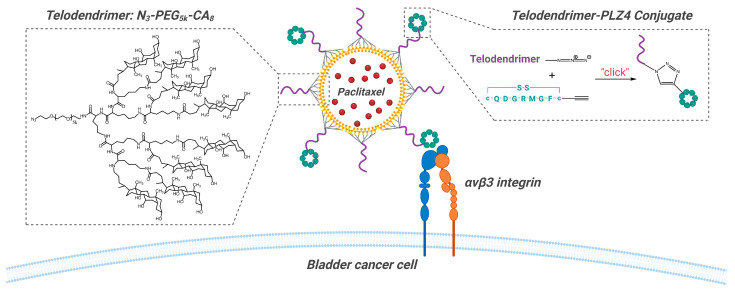
Schematic representation of the structure and targeting mechanism of PPM. The telodendrimers (N_3_-PEG_5k_-CA_8_), featuring a hydrophilic PEG chain and hydrophobic cholic acid dendritic ends, undergo a click reaction with the PLZ4 peptide ligand, to modify the telodendrimer with PLZ4. A 1:1 mixture of telodendrimers, both with and without PLZ4, self-assemble into micelles via thin-film hydration, encapsulating paclitaxel within their core. The PLZ4 ligands on the micelle surface specifically target the αvβ3 integrin expressed on bladder cancer cells, facilitating the targeted delivery of paclitaxel to the tumor site.

PPM (PLZ4-coated paclitaxel-loaded micelle) is a targeted nanoscale (~20 nm) micelle formulation of paclitaxel. PLZ4 is a peptide ligand (cQDGRMGFc) that specifically binds to αvβ3 integrin, which is expressed on bladder cancer cells. The micelles are composed of telodendrimer N_3_-PEG_5k_-CA_8_, consisting of linear polyethylene glycol (PEG) with an azide group at the end and dendritic octamers of cholic acid (CA) block copolymers. PLZ4 is conjugated to the telodendrimers via Huisgen cyclization “click” chemistry [10,11], resulting in micelles that display PLZ4 on their surface (Figure 4). The micelles are prepared using the thin-film hydration method, where the hydrophobic cholic acid forms a core that efficiently loads paclitaxel, achieving a loading efficiency of 99.4% [12]. The safety and effectiveness of PPM are being evaluated in BCG-unresponsive NMIBC patients in an ongoing phase 1 dose escalation trial (NCT05519241). In this study, 50 mL of PPM solution is administered through intravesical instillation once weekly for six weeks. The study is currently recruiting, and no results have been published [13].

### 3.2. Albumin-Based Nanoparticles

Nanoparticle albumin-bound (nab) is an emulsion-based drug delivery technology that involves dissolving a chemotherapeutic agent in an organic solvent and emulsifying it in an aqueous solution of human serum albumin. This primary emulsion is then subjected to high shear force homogenization, resulting in nanoparticles with a hydrophobic core surrounded by a cross-linked albumin shell (Figure 3e) [14]. Nab formulations were developed for improved solubility of poorly water-soluble drugs and the elimination of the need for toxic solvents or dispersants, such as Cremophor EL [15]. Nab-paclitaxel (Abraxane, ~130 nm) is formulated with a 9:1 mass ratio of human albumin to paclitaxel [16] that is administered intravenously in combination with tislelizumab, a humanized IgG4 anti-PD-1 monoclonal antibody, to treat high-risk NMIBC in an ongoing open label, single-arm phase 2 study (TRUCE-02, NCT04730232). Patients receive tislelizumab (IV) 200 mg on day 1, followed by nab-paclitaxel (IV) 200 mg on day 2. This sequential administration is repeated every three weeks for 1 year. Interim results showed a CR rate of 60.7% and an estimated 1-year duration of response (DOR) rate of 88.1%. Treatment-related adverse events (TRAEs) were experienced by 94% of patients, with grade 3–4 TRAEs occurring in 9% of patients [17]. Another example is ABI-009 (nab-rapamycin), formulated with an 8.5:1 mass ratio of human albumin to rapamycin [18], which was administered intravesically in combination with gemcitabine to treat NMIBC in a phase 1/2 study (NCT02009332). In this trial, patients received sequential intravesical instillations once weekly for 6 weeks. ABI-009 suspension (200 mg in 80 mL of 0.9% saline) was administered to the bladder and retained for one hour, followed by gemcitabine (2000 mg in 100 mL saline) administered after voiding of ABI-009 and retained for another hour [19]. Phase 2 results have not been published.

### 3.3. Indwelling Device

TAR-200, also known as GemRIS, is an osmotic pump-based drug delivery system that comprises two main components: (1) a 5 cm semipermeable silicone tube that ensures the sustained release of pre-loaded gemcitabine over three weeks; and (2) incorporated shape memory nitinol wiring within the silicone tube, which allows the device to self-coil into a pretzel shape, thereby preventing its expulsion from the bladder (Figure 3g) [20,21]. According to an ongoing phase 2b clinical study (SunRISe-1, NCT04640623), TAR-200 is placed into the bladder through a urethral catheter on day 0 and is dosed every three weeks for the first 24 weeks, then every 12 weeks through week 99. Interim results showed a CR rate of 83% and an estimated 1-year DOR rate of 75%. However, 72% of the patients experienced TRAEs, with 5% discontinuing the treatment due to their TRAEs [22]. Additionally, a phase 3 randomized, open-label, multi-center study (SunRISe-5, NCT06211764) has recently launched, aiming to evaluate the efficacy and safety of TAR-200 versus single-agent intravesical chemotherapy (MMC or gemcitabine) in the treatment of recurrent high-risk NMIBC after BCG therapy.

## 4. Antibody-Based Therapies and Conjugates

### 4.1. Human/Humanized Monoclonal Antibodies (mAbs)

**Figure 5:**
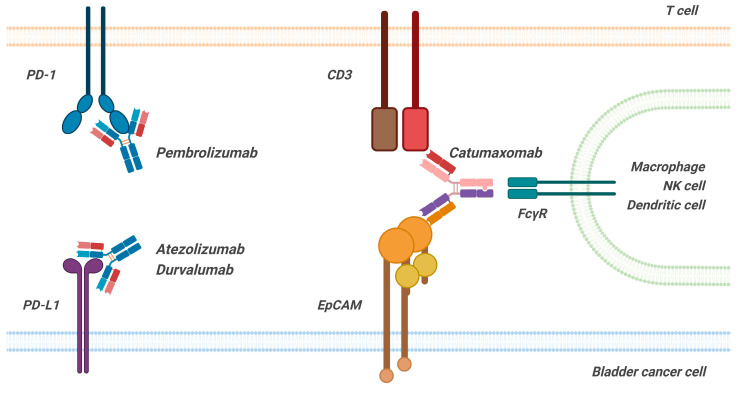
Schematic representation of the mechanisms of action for investigated mAbs in high-risk BCG-unresponsive NMIBC treatment. Human/humanized mAbs (**left**) bind to either PD-1 on T cells (pembrolizumab) or PD-L1 on tumor cells (atezolizumab and durvalumab), preventing the interaction of immune checkpoints. The hybrid mAb catumaxomab (**right**) binds to EpCAM on bladder cancer cells and CD3 on T cells, bringing them into close proximity. Its Fc region also recruits FcγR-positive cells such as macrophages, NK cells, and dendritic cells, enhancing the immune response against bladder cancer cells.

Intravenously administered PD-1/PD-L1 inhibitors have been investigated and approved for the treatment of high-risk BCG-unresponsive NMIBC (Figure 5). These antibodies are administered with either 0.9% sodium chloride or 5% dextrose injection every 3 weeks for up to 2 years [23]. The monotherapy efficacies of pembrolizumab (Keytruda, the only currently FDA-approved PD-1/PD-L1 inhibitor for this indication), atezolizumab, and durvalumab have been evaluated for treating high-risk BCG-unresponsive NMIBC with CIS with or without papillary tumors (Table 1). Additionally, intravenous PD-1/PD-L1 inhibitors are being investigated in combination with intravesical BCG (NCT03711032, NCT02792192, NCT03317158, NCT03519256, and NCT03892642), external radiotherapy (NCT03317158 and NCT03950362), and oral BMS-986205, an IDO1 inhibitor (NCT03519256).

Intravesical PD-1/PD-L1 inhibitors have also been explored, aiming to reduce systemic exposure and associated toxicities. The efficacy and tolerability of intravesical instillation of pembrolizumab in combination with BCG were studied in a phase 1 dose-escalation trial (NCT02808143) in high-risk BCG-unresponsive NMIBC patients. Pembrolizumab was administered intravesically and retained in the bladder for 2 h, involving: (1) a pre-induction phase with a single intravesical dose of pembrolizumab on week -2; (2) a 6-week induction phase with weekly intravesical BCG instillations at weeks 1, 3, and 5, and a combination of intravesical BCG with pembrolizumab at weeks 0, 2, and 4; and (3) a maintenance phase with intravesical pembrolizumab administered every 2 weeks from weeks 7 to 15, and then every 4 weeks from weeks 17 to 49. Five out of the nine patients were still alive at the end of the trial, with a median progression-free survival of 36 months. The 6-month and 1-year recurrence-free rates were 67% (6 of 9) and 22% (1 of 9), respectively. The maximum tolerated dose (MTD) was not reached, but one grade 5 adverse event occurred, resulting in patient death due to myasthenia gravis, which was attributed to pembrolizumab [28].

### 4.2. Hybrid Bispecific Monoclonal Antibody

Catumaxomab is an EMA-approved trifunctional bispecific antibody for the intraperitoneal treatment of malignant ascites. It is a hybrid rat–mouse mAb with an epithelial cellular adhesion molecule (EpCAM) binding arm (mouse IgG2a) and a CD3 binding arm (rat IgG2b), enabling it to simultaneously bind to EpCAM on tumor cells and CD3 on T cells. Additionally, its chimeric mouse/rat Fc region recruits Fc-γ receptor-positive accessory cells, such as macrophages, NK cells, and dendritic cells [29,30,31]. The safety and tolerability of intravesical catumaxomab instillation in high-risk NMIBC patients are being investigated in an ongoing phase 1 open-label dose escalation study (NCT04819399). Catumaxomab is administered in 40 mL PBS solution (pH 7.4) via a transurethral catheter, held in the bladder for at least two hours, with six weekly instillations [30]. Interim results showed a high CR rate and revealed that intravesical catumaxomab instillations are well tolerated in high-risk NMIBC patients, without serious adverse events (SAEs) or systemic exposure [32].

### 4.3. Antibody–Drug Conjugate (ADC)

**Figure 6:**
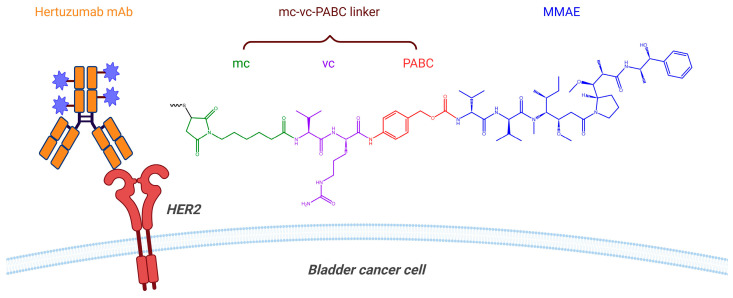
Schematic representation of RC48 structure and its targeting mechanism. Hertuzumab mAb (orange) enables the targeted delivery of MMAE (blue) to HER2 (red)-expressing bladder cancer cells. This is followed by internalization of RC48 and lysosomal cleavage of the mc-vc-PABC linker, releasing the cytotoxic MMAE to exert its therapeutic effect.

RC48 (disitamab vedotin) is an ADC that consists of an anti-HER2 mAb (hertuzumab, also known as disitamab) conjugated to the antimitotic agent monomethyl auristatin E (MMAE) via a cleavable mc-vc-PABC linker [33]. This linker is composed of three parts: (1) mc (maleimidocaproyl), which allows for site-specific conjugation to the cysteine residues of the mAb; (2) valine-citrulline (vc), a dipeptide designed to be recognized and cleaved by cathepsin enzymes in lysosomes; and (3) para-amino benzyloxycarbonyl (PABC), a self-immolative moiety that undergoes a 1,6-elimination, releasing MMAE as the active cytotoxic agent (Figure 6) [34,35]. The efficacy and safety of RC48 in combination with tislelizumab are being evaluated in an ongoing open-label phase 2 clinical trial (TRUCE-04, NCT05495724) in patients with HER2 overexpressing high-risk NMIBC that is not completely resectable. Patients receive 120 mg of RC48 intravenously on day 1 and 200 mg of tislelizumab intravenously on day 2 every three weeks for 3 or 4 cycles. Interim results based on 24 patients showed a CR rate of 66.7% (16 of 24), with 33.3% (8 patients) receiving RC48 monotherapy and 66.7% (16 patients) receiving RC48 combined with tislelizumab. Grade 3–4 TRAEs were reported in 16.7% of patients (4 of 24) [36].

## 5. Recombinant Fusion Protein

### 5.1. Cytokine–Fusion Protein Complex

**Figure 7:**
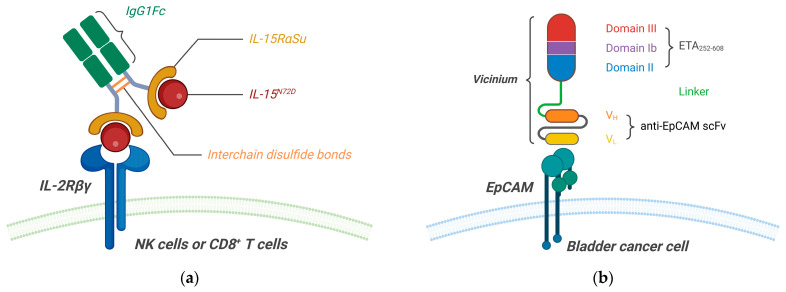
Schematic representation of the structures and targeting mechanisms of investigated recombinant fusion proteins in high-risk BCG-unresponsive NMIBC treatments. (**a**) Anktiva consists of two 114-amino acid (aa) IL-15N72D variants bound to a dimeric IL-15RαSu/Fc. The IL-15N72D (nogapendekin alfa) binds to the 65-aa IL-15RαSu, and the IL-15RαSu/Fc dimerizes to form inbakicept via interchain disulfide bonds between the two 232-aa Fc domains [39]. This IL-15N72D is presented in trans to the IL-2Rβγ complex on NK cells and CD8+ T cells, promoting their activation and proliferation without stimulating Tregs. (**b**) Vicinium is a scFv-toxin fusion protein that consists of an anti-EpCAM scFv (VH region in orange and VL region in yellow) fused to a truncated form of Pseudomonas exotoxin A (ETA252-608: domain II in blue, domain Ib in purple, and domain III in red) via a 20-aa peptide linker (EFGGAPEFPKPSTPPGSSGL) [40]. The scFv component enables targeted delivery of Vicinium to EpCAM expressed on bladder cancer cells.

Anktiva (also known as ALT-803, N-803, or nogapendekin alfa inbakicept-pmln) is a recently FDA-approved interleukin-15 (IL-15) receptor agonist indicated for use in combination with BCG to treat BCG-unresponsive NMIBC with CIS with or without papillary tumors. The human IL-15^N72D^ variant (nogapendekin alfa) and the human IL-15Rα sushi domain/human IgG1 Fc (IL-15RαSu/Fc) fusion protein are co-expressed by Chinese hamster ovary (CHO) cells. The IL-15^N72D^ binds to the IL-15Rα sushi domain on IL-15RαSu/Fc, which then dimerizes via interchain disulfide bonds of the Fc region, forming inbakicept. Each fully assembled Anktiva complex consists of two nogapendekin alfa components bound to a single inbakicept molecule (Figure 7a) [37]. This complex specifically targets the IL-2Rβγ complex on NK cells and CD8^+^ T cells, promoting their activation and proliferation without stimulating regulatory T cells (Tregs) [38]. The efficacy and safety of Anktiva with BCG were evaluated in a registrational single-arm, multicenter, phase 2/3 trial (QUILT-3.032, NCT03022825). Anktiva 400 mcg is combined with BCG (50 mg in 50 mL of saline), then administered intravesically and retained in the bladder for 2 h. This process is repeated weekly for six weeks as induction therapy, with additional maintenance doses administered at specified intervals for up to 37 months. The efficacy results showed that 62% of the patients (48 of 77) achieved a CR at any time, with a 12-month DOR of 58% and a 24-month DOR of 40%. Serious adverse reactions occurred in 16% of patients (14 of 88), with hematuria (3.4%), urinary tract infection (2.3%), and musculoskeletal pain (2.3%) being the most common grade 3/4 adverse reactions. One patient (1.1%) experienced a fatal cardiac arrest [39].

### 5.2. Single-Chain Variable Fragment (scFv)-Targeted Toxin

Vicinium, also known as oportuzumab monatox or VB4-845, is an *Escherichia coli*-expressed recombinant fusion protein composed of 648 amino acids. It consists of a humanized anti-EpCAM scFv tethered to a truncated form of Pseudomonas exotoxin A (ETA_252-608_) via a 20 amino acid peptide linker (Figure 7b) [40,41]. The efficacy of Vicinium as a monotherapy for treating high-risk BCG-unresponsive NMIBC was evaluated in an open-label, single-arm, multicenter phase 3 trial (VISTA, NCT02449239). Vicinium (30 mg/50 mL saline) was administered intravesically twice weekly for 6 weeks, followed by once weekly for another 6 weeks as induction therapy, then once weekly every other week for up to 104 weeks as maintenance therapy. For CIS patients, the 3-month CR rate was 40% (36 of 89), with a 24-month disease-free rate of 31% (11 of 36). For papillary patients, the 3-month recurrence-free rate was 71% (27 of 38), with a 24-month disease-free rate of 44% (12 of 27) [42]. Moreover, the combination of intravesical Vicinium with intravenous durvalumab was evaluated in a phase 1 single-arm clinical trial (NCT03258593) in BCG-unresponsive, high-risk NMIBC patients. Durvalumab 1500 mg was administered intravenously once every 4 weeks for 12 or 24 months, then once every 3 months during the second year for maintenance therapy. Vicinium (30 mg/50 mL saline) was administered intravesically once weekly for 12 weeks, followed by every other week for up to 2 years. Final results showed disease-free rates of 47% (7 of 15 at 12 weeks), 40% (6 of 15 at 6 months), and 20% (3 of 15 at 12 months). All patients experienced TRAEs, 27% of which were grade 3 or higher. Median recurrence-free survival was 13.2 months [43]. The biologics license application for Vicinium was denied by the FDA in 2021.

## 6. Nucleic Acid Therapeutics

### 6.1. Adenoviral Vectors

Adstiladrin (nadofaragene firadenovec-vncg) is an FDA-approved, non-replicating adenoviral vector-based gene therapy for the treatment of high-risk BCG-unresponsive NMIBC with CIS with or without papillary tumors. It is a recombinant adenovirus serotype 5 vector (E1 and E3 genes deleted to prevent viral replication) containing human IFNα2b complementary DNA (Figure 3b) [44,45]. The adenovirus interacts with the coxsackie/adenovirus receptor (CAR) on urothelial cells, leading to CAR-mediated endocytosis and sustained production of IFNα2b in the urine for up to 12 days [45,46]. This formulation is provided as a sterile frozen suspension for intravesical instillation, with a nominal concentration of 3 × 10^11^ viral particles (vp)/mL. To preserve the viability of the live virus in the suspension, the formulation includes cryoprotectants (glycerol and sucrose), stabilizers (hydroxypropyl-β-cyclodextrin, polysorbate 80, and MgCl_2_·6H_2_O), and buffering agents (HOC(CH_2_CO_2_H)_3_·H_2_O, HOC(CH_2_CO_2_Na)_3_·2H_2_O, NH_2_C(CH_2_OH)_3_, and NaH_2_PO_4_·2H_2_O). Additionally, 0.95 mg/mL of [N-(3-cholamidopropyl)-N-(3-lactobionamidopropyl)]-cholamide (Syn3), a polyamide surfactant, is included to enhance adenoviral transduction across the GAG layer of the bladder lining [46]. The efficacy and safety of Adstiladrin were both evaluated in a single-arm, open-label, multicenter phase 3 clinical trial (CS-003, NCT02773849). Adstiladrin (75 mL) was administered intravesically once every three months for up to 12 months. The CR rate was 51% by 3 months, with a median DOR of 9.7 months, and 46% of the responders maintained a CR for at least one year. Serious adverse reactions occurred in 11% of patients, and 1.9% of patients discontinued treatment permanently due to adverse reactions [46]. According to a 5-year follow-up, 5.8% of patients with CIS and 15% of patients with high-grade Ta/T1 were high-grade recurrence-free (HGRF) at month 57. The overall survival and cystectomy-free survival rates at 60 months were 80% and 49%, respectively, with only 5 patients experiencing clinical progression to muscle-invasive disease [47]. 

CG0070 (cretostimogene grenadenorepvec) is a conditionally replicating adenovirus encoding the complementary DNA for an immunogenic cytokine, human granulocyte– macrophage colony–stimulating factor (GM-CSF). The engineered serotype 5 adenovirus (Ad5) provides selective viral replication and cytokine expression in tumor cells, controlled by a dual-promoter system: (1) E2F-1, a tumor-specific promoter, drives the expression of E1A, which is essential for viral replication leading to tumor cell lysis; and (2) E3, a promoter activated by E1A gene products that control the tumor-specific expression of GM-CSF to induce an anti-tumor immune response (Figure 3b) [48]. Following the observation of a tolerability safety profile assessment in a phase 1 dose-escalation study [49], the efficacy of CG0070 in combination with pembrolizumab was evaluated in a phase 2 single-arm, open-label clinical trial (CORE-001, NCT04387461). A mild detergent, 0.1% n-dodecyl β-D-maltoside (DDM), and 1 × 10^12^ vp of cretostimogene in 100 mL of saline were administered intravesically once a week for 6 or 12 weeks, followed by three weekly maintenance instillations at months 3, 6, 9, 12, and 18. Pembrolizumab (400 mg) was administered intravenously every 6 weeks for up to 18 administrations. Final results showed a 12-month CR rate of 57.1% (20 of 35) and a 24-month CR rate of 51.4% (18 of 35), with progression-free survival (PFS) of 100% at 24 months [50]. As a monotherapy, the efficacy of CG0070 for the treatment of high-risk BCG-unresponsive NMIBC is being evaluated in an ongoing phase 3, single-arm clinical trial (BOND-003, NCT04452591). The dosing regimen of CG0070 is identical to that in CORE-001. Interim data showed a CR rate at any time of 75.2% (79 of 105) and a 12-month DOR rate of 83% [51].

### 6.2. Plasmid Polyplex

BC-819 (inodiftagene vixteplasmid) is a recombinant DNA plasmid encoding a potent cellular toxin, diphtheria toxin A (DTA), complexed with a transfection reagent polyethylenimine (PEI). The expression of DTA is controlled by the tumor-specific H19 promoter, ensuring that DTA is only expressed in malignant cells and not in normal tissues (Figure 3c) [52]. The efficacy of BC-819 monotherapy in treating BCG-unresponsive NMIBC was evaluated in a phase 2, open-label, single-arm, multicenter clinical trial (NCT03719300). Patients received 20 mg of BC-819 in a total volume of 50 mL intravesically, once weekly for 10 weeks during the induction phase, followed by maintenance therapy every 3 weeks for up to 84 weeks. This trial was terminated early due to a lack of efficacy: the CR rate at week 12 was 17.6% (3 of 17) [53]. 

**Figure 8:**
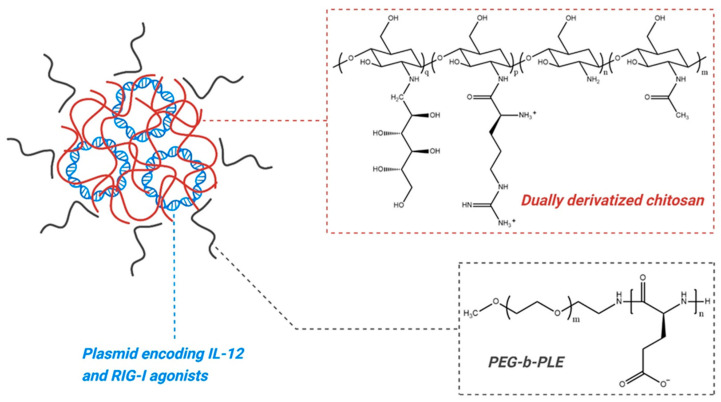
Schematic representation of the EG-70 delivery system. The core is a polyplex formed by plasmids (blue) complexed with derivatized chitosan polymers (red), which are functionalized with arginine and glucose. This positively charged polyplex is reversibly coated with the polyanion-containing PEG-b-PLE (black).

EG-70 (detalimogene voraplasmid) is a non-viral gene-based immunotherapy developed using enGene’s Dually Derivatized Oligochitosan (DDX) platform. It comprises the following: (1) a plasmid encoding both recombinant human interleukin-12 (IL-12) and two double-stranded RNA agonists (eRNA1 Ia and VA1) that activate the retinoic acid-inducible gene I (RIG-I) pathway; (2) a dually derivatized chitosan polymer functionalized with arginine and glucose, which complexes with the plasmid; and (3) a polyethylene glycol–polyglutamic acid (PEG-b-PLE) polymer, which forms an electrostatically assembled coating around the plasmid-DDX polyplex [54] (Figure 8). The safety, tolerability, and efficacy of EG-70 in treating high-risk BCG-unresponsive NMIBC with CIS are being evaluated in a phase 1/2, open-label, multicenter clinical trial (LEGEND, NCT04752722). The phase 1 portion followed a 3 + 3 dose escalation design, where patients received 2 or 4 intravesical doses of EG-70 over a 12-week period at dose levels of 0.25 mg/mL, 0.8 mg/mL, and 2.5 mg/mL. A total of 73% of patients (16 of 22) achieved a CR at any time; 54.2% of patients (13 of 24) experienced TRAEs, with 12 being grade 1/2 and 1 being grade 3. The recommended phase 2 dose (RP2D) was established as intravesical administration of EG-70 (0.8 mg/mL in 50 mL solution, retention time 60 min) on weeks 1, 2, 5, and 6 of a 12-week cycle. At RP2D, phase 1 CR rates were 70% (7 of 10) at 3 months and 60% (6 of 10) at 6 months, with a 3-month DOR of 86% (6 of 7) and a 6-month DOR of 75% (3 of 4) [55]. The phase 2 portion of the LEGEND study is ongoing.

### 6.3. Oligonucleotide–Lipid Conjugate

RAG-01 is a small activating RNA (saRNA) designed to activate the tumor suppressor gene p21 [56]. This duplex RNA is delivered by a proprietary lipid-conjugated oligonucleotide (LiCO™) delivery system, consisting of a lipid conjugated to duplex RNA by a benzimidazole linker (Figure 3d). The safety and preliminary efficacy of RAG-01 in NMIBC patients who have failed BCG therapy are being evaluated in a recently launched phase 1, open-label, multi-center study (NCT06351904). RAG-01 is administered intravesically, with initial cohorts receiving a starting dose of 30 mg, followed by dose-escalation cohorts receiving 100 mg, 300 mg, and 600 mg, respectively [57]. Specific volumes and frequency of administration, unfortunately, have not been disclosed. 

## 7. Discussion

Recent efforts toward developing novel therapies for high-risk BCG-unresponsive NMIBC have initially focused on the intravenous administration of PD-1/PD-L1 inhibitors, marked by the approval of KEYTRUDA in 2020. However, to reduce systemic exposure and associated toxicities, attention has shifted to intravesical instillation (Table 2). This approach directly targets the bladder tumor, minimizing systemic side effects and maximizing local drug concentration. 

Given the limited administration frequency possible with catheter-based delivery approaches, a notable trend in intravesical therapy has been directed toward extending the contact time between the therapeutic agent and the tumor. One promising method is TAR-200, an indwelling device that provides sustained drug release over several weeks, showing positive outcomes in clinical trials. However, the need for surgical removal of the device after treatment raises significant safety and compliance concerns. Another strategy involves gene therapies, such as Adstiladrin, CG0070, and EG-70, that deliver cytokine-expressing genes and generate therapeutic agents from urothelial cells over time. 

Gene therapies have gained traction as potential treatments for high-risk BCG-unresponsive NMIBC, with several receiving fast track and/or breakthrough therapy designations from the FDA (Figure 2). Putative transduction-enhancing excipients, such as Syn3 in Adstiladrin and DDM in CG0070, are believed to facilitate penetration across the bladder GAG layer. Additionally, RAG-01 utilizes a conjugated lipid to penetrate the GAG layer, underscoring the potential importance of detergent components to enhance gene delivery efficiency, either as excipients or integrated into the delivery system. Another important aspect of gene therapies is selective delivery and/or activation to specifically impact their function within tumor cells. For example, the INFα2b encoded in Adstiladrin is expressed by both tumor cells and surrounding epithelial cells; however, the tumor-specific expression of the therapeutic gene product is controlled by promoters in CG0070 and BC-819. Although these therapeutics are delivered non-selectively, the promoter-driven expression minimizes off-target effects and maximizes therapeutic efficacy. It is worth noting that CG0070 showed a markedly improved safety profile over that of Adstiladrin, which may potentially be linked to its selectivity.

Several key clinical trials in the next five years will likely shape the future landscape of high-risk BCG-unresponsive NMIBC treatments. SunRISe-5 (NCT06211764) is the first randomized trial in this indication, providing a head-to-head comparison between the sustained drug release from TAR-200 and traditional intravesical chemotherapy. It is expected to provide robust evidence on the risks, benefits, and treatment durability of extended drug interaction time. BOND-003 (NCT04452591) focuses on CG0070 monotherapy and includes the largest sample size (N = 112) ever in this indication, with interim efficacy and safety results showing promising outcomes that potentially surpass the three approved drugs. NCT06351904 represents the first RNA conjugate-based gene delivery system for intravesical treatment (RAG-01). This agent received an FDA Fast Track designation within two months after the first patient was dosed. This trial will provide critical insights into the stability of the lipid-conjugated delivery approach in the human bladder environment and the efficacy of its GAG-penetrating strategy, which links GAG-destructive components directly to the therapeutic substance, unlike previous methods that use separate detergents as excipients. These trials are expected to provide crucial data that could revolutionize the treatment strategies for high-risk BCG-unresponsive NMIBC and offer new hope for patients seeking bladder-preserving therapies.

## Figures and Tables

**Table 1:** Monotherapy of PD-1/PD-L1 inhibitors for high-risk BCG-unresponsive NMIBC.

Drug	Trial ID	CR	DOR	TRAEs
Pembrolizumab * [24]	KEYNOTE-057 NCT02625961 Phase 2	3-Month CR rate = 41% (39 of 96)	Median DOR = 16.2 Mo DOR ≥ 12 Mo = 46% (18 of 39)	Grade 3–4 = 13% (13 of 101) Serious TRAEs = 8% (8 of 101)
Atezolizumab [25]	SWOG S1605 NCT02844816 Phase 2	6-Month CR rate = 27% (20 of 74)	Median DOR = 17 Mo DOR ≥ 12 Mo = 56% (11 of 20)	Grade 3–5 = 16% (26 of 166)
Atezolizumab [26]	NCT02792192 Phase 1b/2	6-Month CR rate = 33% (4 of 12)	Median DOR = 6.8 Mo	Grade 3 = 25% (3 of 12)
Durvalumab [27]	ADAPT-BLADDER NCT03317158 Phase 1/2	3-Month CR rate = 33% (1 of 3)	DOR ≥ 12 Mo = 0% (0 of 1)	Not reported

* FDA approved.

**Table 2:** Summary of FDA-approved drugs for high-risk BCG-unresponsive NMIBC.

	KEYTRUDA	ADSTILADRIN	ANKTIVA
Approval Year	2020	2022	2024
Drug Substance	mAb	DNA Plasmid	Recombinant Fusion Protein
Mechanism	PD-1 Checkpoint Inhibitor	Secreting IFNα2b	IL-15 superagonist
RoA	Intravenous Injection	Intravesical Instillation	Intravesical Instillation
Delivery System	N/A	Adenoviral Vector	Fusion Protein Complex
Clinical Trial	NCT02625961 (KEYNOTE-057)	NCT02273849 (CS-003)	NCT03022825 (QUILT-3.032)
CR Any Time	41% (39/96)	51% (50/98)	62% (48/77)
DOR ≥ 12 Month	46% (18/39)	46% (23/50)	58% (28/48)

## List of Abbreviations

ADC	antibody–drug conjugate
Ad5	serotype 5 adenovirus
BCG	Bacillus Calmette–Guérin
BsAb	bispecific antibody
CA	cholic acid
CAR	coxsackie/adenovirus receptor
CHO	Chinese hamster ovary
CIS	carcinoma in situ
CR	complete response
DDM	n-Dodecyl β-D-maltoside
DDX	dually derivatized oligochitosan
DOR	duration of response
DTA	diphtheria toxin A
EpCAM	epithelial cellular adhesion molecule
GAG	glycosaminoglycan
GM-CSF	granulocyte–macrophage colony-stimulating factor
HG	high grade
HGRF	high-grade recurrence-free
IL-15	interleukin-15
LG	low grade
LVI	lymph vascular invasion
mAb	monoclonal antibody
MIBC	muscle-invasive bladder cancer
MMAE	monomethyl auristatin E
MTD	maximum tolerated dose
Nab	nanoparticle albumin-bound
NMIBC	non-muscle invasive bladder cancer
PABC	para-amino benzyloxycarbonyl
PEI	polyethylenimine
PEG	polyethylene glycol
PEG-b-PLE	polyethylene glycol–polyglutamic acid
PPM	PLZ4-coated paclitaxel-loaded micelle
PUNLMP	papillary urothelial neoplasm of low malignant potential
RC	radical cystectomy
RIG-I	retinoic acid-inducible gene I
RoA	route of administration
RP2D	recommended phase 2 dose
saRNA	small activating RNA
SAEs	serious adverse events
scFv	single-chain variable fragment
Tregs	regulatory T cells
Syn3	[N-(3-cholamidopropyl)-N-(3-lactobionamidopropyl)]-cholamide
TRAEs	treatment-related adverse events
TURBT	transurethral resection of bladder tumor
vc	valine-citrulline
vp	viral particles
